# Efficient Control of THz Transmission of PEDOT:PSS with Resonant Nano-Metamaterials

**DOI:** 10.1038/s41598-019-54189-9

**Published:** 2019-11-27

**Authors:** Raghvendra P. Chaudhary, Bamadev Das, Seugn In Oh, Dai-Sik Kim

**Affiliations:** Department of Physics and Astronomy and Center for Atom Scale Electromagnetism, Seoul National University, Seoul, 08826 Republic of Korea

**Keywords:** Metamaterials, Optical manipulation and tweezers

## Abstract

Nano-metamaterials designed to operate at a certain resonance frequency enhance the magnitude of terahertz (THz) wave transmission by three orders of magnitude or even more. In this pursuit, controlling magnitude of resonant transmission and tuning the resonance frequency is increasingly important for application in low power THz electronics and devices. THz optical properties of chemically doped poly(3,4‐ethylenedioxythiophene):poly(4‐styrenesulfonate) (PEDOT:PSS) have been studied, however its effect on the THz transmission properties in combination with nano-metamaterials have not yet been demonstrated. Here we demonstrate the efficient control over resonant THz transmission and tuning of resonance frequency of different nano-metamaterials using PEDOT:PSS, without any toxic chemical doping. By ease of simple solution processing with single step and drop-casting 10 μL aqueous solution of PEDOT:PSS on different nano-metamaterials with varied concentrations, we were able to dynamically control the THz transmission along with resonance frequency. This dynamic control of transmission and shift in resonance frequency can be attributed to improved conductivity of PEDOT:PSS and its interaction with strongly localized THz field of the metamaterial.

## Introduction

Functional terahertz (THz) devices have recently gained renown interest due to their wide range of applications in modulators, electro-optic devices, filters, wireless communication, THz spectroscopy & imaging, biomedical applications etc^1–6^. Several attempts have been made to manipulate THz wave using semiconducting metamaterials^7–10^, electrically modulating the intraband transition in graphene^11–13^, thermally induced phase change in VO_2_^14–17^ and doped conducting polymers^18–20^. Poly(3,4-ethylenedioxythiophene):poly(4-styrenesulfonate) (PEDOT:PSS) as a π-conjugated conducting polymer has high thermal stability, high transparency and tunable opto-electronic properties^21,22^. The conductivity properties of PEDOT:PSS could be tuned by chemical doping and has been studied using THz spectroscopy^20,23^. Also, doped PEDOT:PSS has been employed as a THz beam splitter, broadband THz antireflection coating, electrically tunable THz liquid phase shifter, efficient hole transport layer etc^24–26^. One of the advantages of PEDOT:PSS is its ease of solution processing and handling^27^. To date the effective control of THz in nano-metamaterials with the combination of cost effective techniques and nontoxic chemicals is still void in literature and needs to be explored. Also most of the reported nano-metamaterials work passively, in which the frequency response depends on their physical parameter.

Herein we report on control of THz transmission and tuning of resonance frequency using PEDOT:PSS with nano-metamaterial structures without any toxic chemical doping. Effective THz transmission of PEDOT:PSS coated sub-wavelength rectangular ring nanogap based antenna and slot antennas have been demonstrated. Different weight percentage (wt%) of aqueous PEDOT:PSS solution was drop casted over the antenna arrays forming a thin film. These PEDOT:PSS film coated resonant antenna arrays were utilized for THz transmission measurements. With increasing the PEDOT:PSS concentration, THz transmission through the antenna array was found to reduce, implying controlled transmission of the incident THz. Also red shift in the resonance frequency of the antenna was observed with increasing the concentration of PEDOT:PSS. This effective control over transmission and tuning of resonance frequency is a result of the enhanced extinction ratio of PEDOT:PSS due to strong THz field enhancement and localization effects^28,29^. We show that by ease of fabrication and single step solution processing of PEDOT:PSS, we can dynamically control the resonant THz transmission through nano-metamaterial and tune its resonance frequency.

## Experimental Methods

Resonant THz nano-metamaterials based on 20 nm gap width and slot antenna array were used to study the transmission characteristics due to strong THz field enhancement and localization. The array of 20 nm nanogap metamaterial structure extended over rectangular dimension of 20 μm × 80 μm (w × l) and 100 nm deep nanotrenches were fabricated by atomic layer lithography reported elsewhere^30^. The schematic of the fabrication processes of resonant metamaterial structure is presented in figures below. For 20 nm gap fabrication, large area rectangular metallic arrays were defined by UV photolithography in an image reversal photoresist (AZ5214) followed by metal evaporation and lift-off process. Next, conformal 20 nm thin film of aluminium oxide (Al_2_O_3_) was deposited using atomic layer deposition. Subsequently, a second layer of metal deposition defines the vertically aligned metal-Al_2_O_3_-metal interfaces, where Al_2_O_3_ thickness defines the gap width. Since the Al_2_O_3_ is weakly bond to metal layer, excess of the second metal layer was exfoliated using scotch tape and the surface was milled using ion beam milling at a glided angle to flatten the gap surface. For slot antenna fabrication, a negative tone photoresist ma-N2405 (MicroChem) was spin coated (4000 rpm) on Si substrate and soft baked at 120 °C for 120 sec. The array of rectangular pattern of desired dimensions was written using electron beam and developed in ma-D532 developer. Further, the metal evaporation and lift off processes were used to obtain the metamaterial structure of the slot nano-antenna array.

Control of THz in different nano-metamaterials using different wt% of aqueous PEDOT:PSS solution was demonstrated using THz time domain spectroscopy(THz-TDS). The aqueous solution of PEDOT:PSS (pristine solution) from Sigma Aldrich with concentration of 1.3 wt% in H_2_O, where PEDOT and PSS contents are 0.5 wt% and 0.8 wt% respectively were used as received. The pristine solution of PEDOT: PSS was diluted with DI water to get the desired concentration of 0.01 wt%, 0.05 wt%, 0.10 wt% and 0.50 wt%. Before using, the solutions were stirred for 12 hours at room temperature to improve the uniformity. Due to different concentrations, the thickness of the drop-casted films was expected to be different and measured by cross-sectional scanning electron microscopy (SEM). The prepared films were annealed at 150 °C for 15 min to improve the crystallinity. Furthermore, to study the effect of ethylene glycol (EG) doping on THz transmission through the metamaterial structures, pristine solution of PEDOT: PSS was doped with 3 wt% of EG and drop casted in similar manner. EG doping was done to prove that the conductivity enhancement plays the major role in controlling the THz transmission^19^. All the measurements were performed on single device after washing off with acetone and DI water each time.

## Results and Discussion

**Figure 1:**
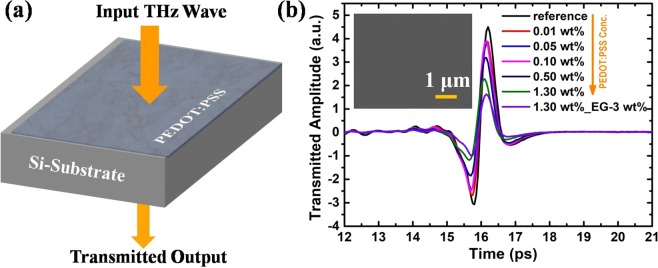
(**a**) Schematic diagram of the THz transmission measurement through PEDOT:PSS coated Si substrate. (**b**) Time domain spectrum of the THz electric field transmitted through different concentrations of PEDOT:PSS coated Si substrate.

First, we measured the broadband THz transmission through PEDOT:PSS drop casted on bare silicon (Si) substrate in the time domain as shown in figure 1(a). Time domain THz spectra, represented in figure 1(b) shows that by increasing the PEDOT:PSS concentration, the THz transmission gets reduced by ~45% for 1.3 wt% PEDOT:PSS compared to reference sample (bare Si). The thickness of the coated PEDOT: PSS film was measured to be ~455 nm for 1.3 wt% concentration and is postulated to be lower for lower concentrations of PEDOT:PSS. Inset in figure 1(b) represents SEM image of coated PEDOT: PSS film and was found to be uniform over the coated area. These results imply the single step and effective control of THz transmission by changing the PEDOT:PSS concentration compared to 2D materials introduced earlier^31,32^, which have been used to study the shielding effect and also require multistep synthesis process. PEDOT:PSS has conducting nature due to presence of PEDOT molecules. The reduction in broadband THz intensity can be attributed to the change in the carrier density of PEDOT:PSS with change in PEDOT to PSS concentration. With increase in the concentration, the aggregation of the PEDOT and PSS particles increases which leads to the increased conductivity, which in turn leads to the reduced THz transmission^20^. The addition of 3 wt% of ethylene glycol (EG) gives rise to improved carrier mobility and hence conductivity, which affects the THz transmission more significantly.

**Figure 2:**
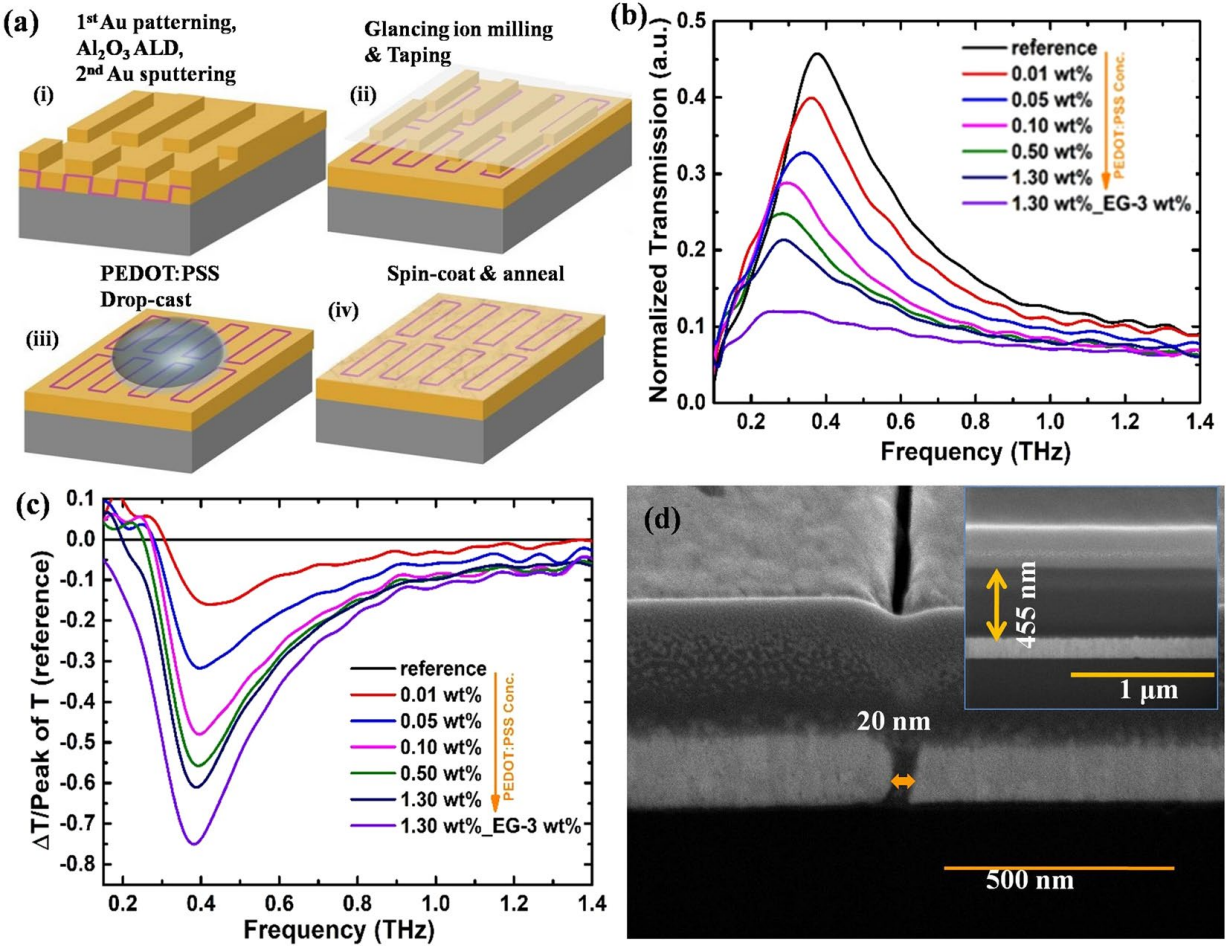
(**a**) Process flow to fabricate large area PEDOT:PSS coated 20 nm nanogap metamaterial. a(i) array of rectangular slits patterned on 1 cm^2^ Si wafer via photolithography followed by Au sputtering and lift off. Al_2_O_3_ film was then deposited conformally on the patterned rectangular array of slit using ALD. The second layer of Au film is sputtered on top of the Al_2_O_3_ coated array of slits. a(ii) Glancing angle ion milling and taping removes extra metal layer on top, exposing Al_2_O_3_ filled nanogap. a(iii) 10 μL drop of PEDOT:PSS was drop-casted on top of the nanogap metamaterial structure. a(iv) thin film of PEDOT:PSS formed after annealing at 150 °C on hot plate to increase the crystallinity. (**b**) Normalized THz transmission spectrum for the nanogap metamaterial structure with different wt% of PEDOT: PSS coating. (**c**) Calculated transmission depth at different concentrations of PEDOT: PSS. A large transmission depth of ~75% was obtained for 3 wt% EG doped PEDOT: PSS. (**d**) Cross section image showing successful fabrication of 20 nm nanogap. Inset shows the thickness of ~455 nm for 1.3 wt% PEDOT: PSS film coated on Au film.

THz transmission properties of PEDOT:PSS through resonant nano-metamaterial structures such as nanogap (20 nm) and nanoslot antenna were also investigated. The measurements were conducted on films of different concentrations of PEDOT:PSS and converted to frequency domain using fast Fourier transformation (FFT). The transmission spectra through the PEDOT:PSS coated metamaterial structure were normalized by the transmission spectrum through the Si substrate. The normalized transmission through uncoated nanogap metamaterial structures is represented as the reference spectrum in the respective figures. Figure 2(a) is the schematic of the nanogap fabrication process. The fundamental resonance frequency of 20 nm nanogap metamaterial is at 0.4 THz and the resonant transmission was found to be reduced with increase in the PEDOT: PSS concentration as shown in figure 2(b). Also the resonance frequency is red shifted with increasing in concentration. This transmission change and shift in resonance frequency can be attributed to the strong enhancement and localization of THz field through the metamaterial system and its interaction with the conducting PEDOT:PSS. The localized THz field can lead to change in the local dielectric environment at the near-field, which results in a red shift and affects the resonant transmission^28,33^. For the 3 wt% EG doped PEDOT:PSS coated nanogap metameterial, THz transmission is drastically reduced due to increase in its conductivity (charge density). A large change in transmission depth of ~75% was observed for 3 wt% EG doped PEDOT:PSS compared to 1.3 wt% PEDOT:PSS (~62%) as shown in figure 2(c). The transmission depth is defined as;

$$\frac{\Delta T}{{T}_{ref}(peak)}=\frac{{T}_{PEDOT:PSS(wt{\rm{ \% }})}-{T}_{ref}}{{T}_{ref}(peak)}$$

Where ${T}_{PEDOT:PSS(wt \% )}$ and ${T}_{ref}(peak)$ are the normalized transmission through PEDOT:PSS coated metamaterial and peak value of transmission for uncoated metamaterial respectively A negligible phase change was observed with increase in the PEDOT:PSS concentration.

**Figure 3:**
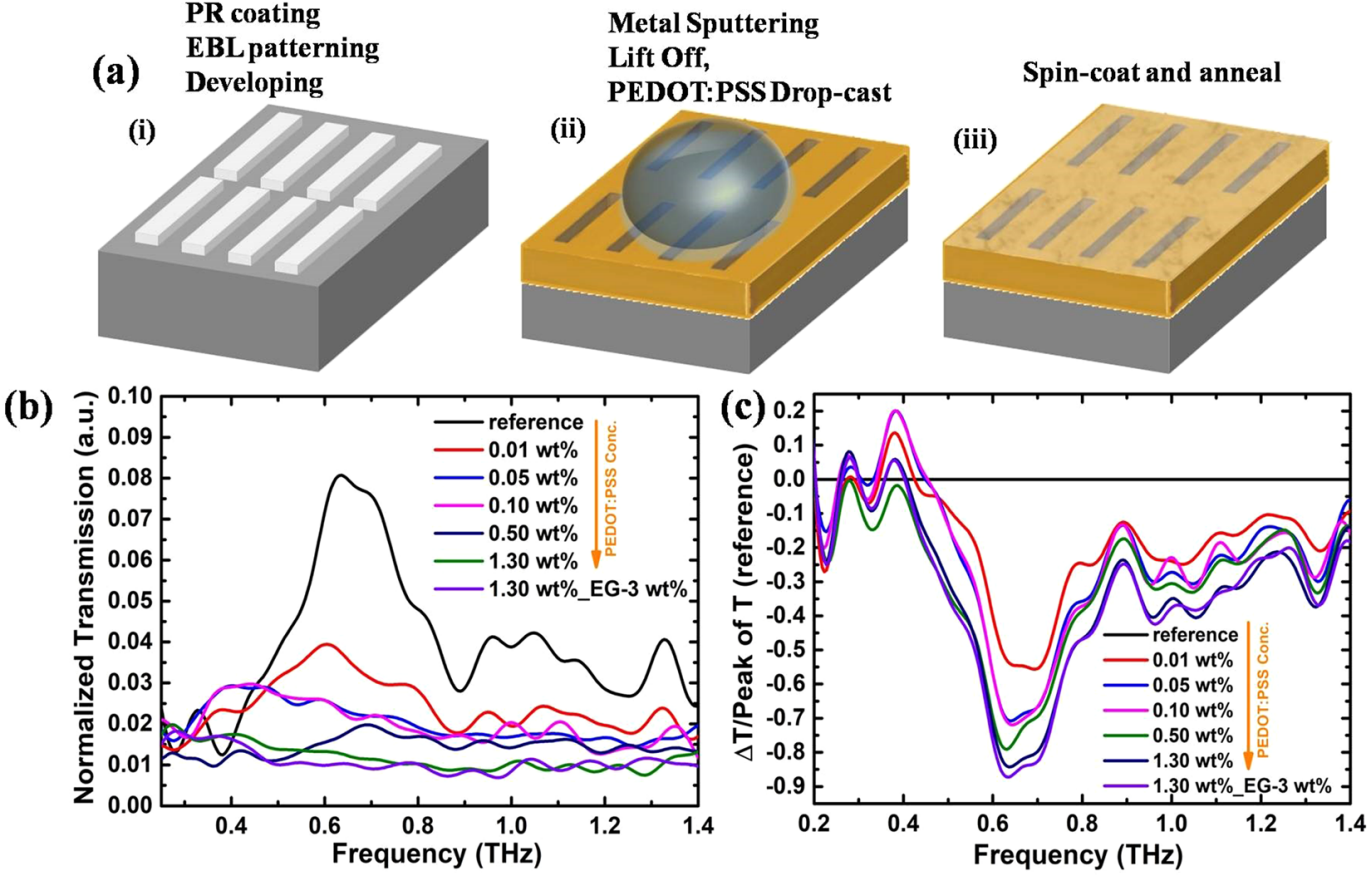
(**a**) Schematic of fabrication process of PEDOT: PSS coated nanoslot antenna. a(i) array of rectangular slits defined in a negative tone resist via electron beam. a(ii) Au sputtering and lift off lead to the formation of nanoslots on which 10 μL of PEDOT: PSS was drop-casted. a(iii) PEDOT:PSS drop was annealed at 150 °C for 15 min to form a thin film. (**b**) Normalized THz transmission spectrum for the 200 nm slot antenna metamaterial coated with different wt% of PEDOT:PSS. (**c**) Calculated transmission depth of the nanoslot antenna for different wt% of PEDOT:PSS. 0.05 wt% of PEDOT:PSS film was sufficient enough to suppress the resonant behavior.

**Figure 4:**
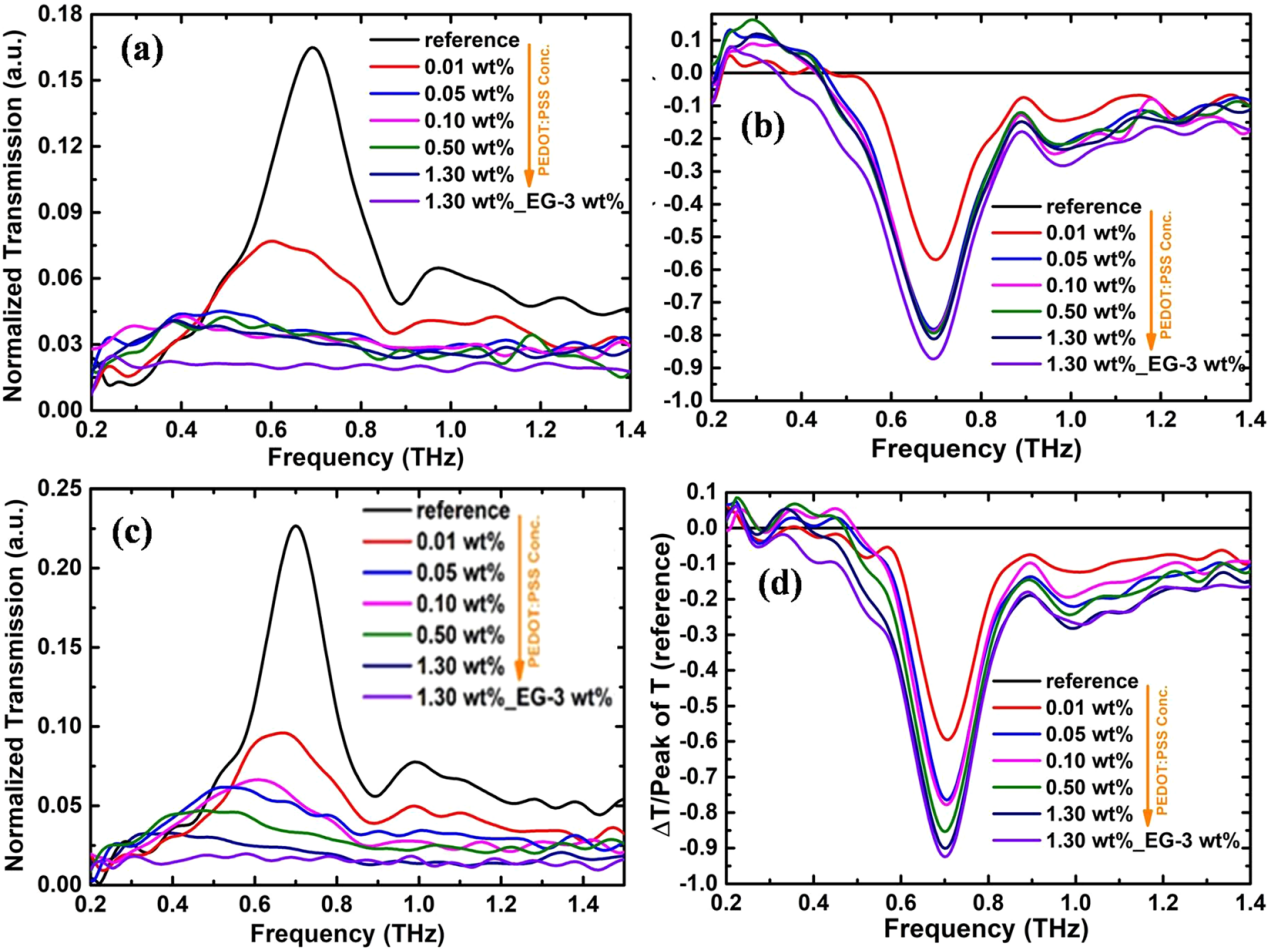
(**a**) Normalized THz transmission spectrum for the 500 nm slot antenna metamaterial coated with different wt% of PEDOT: PSS. **(b**) Calculated transmission depth of the nanoslot antenna. (**c**,**d**) corresponding transmission spectrum and transmission depth calculated for 1000 nm slot antenna.

The THz transmission characteristics of the PEDOT: PSS coated resonant nanoslot antennas are shown in figures 3 and 4. Rectangular nanoslots with 80 μm in length and three different widths of 200, 500 and 1000 nm with fundamental resonance frequency at ~0.7 THz were fabricated. The lateral periodicity was kept 50 μm in each case. With increasing the slot width the field enhancement has been found to reduce compare to 20 nm nanogaps, however field localization is increased to large distance in the surrounding environment^34^. The normalized transmission spectra in the frequency domain are represented in figure 3(b) for 200 nm slot antenna. Compared to 20 nm nanogap based metamaterial, the nanoslot antenna showed drastic change in the transmission properties. The fundamental resonance frequency in this case almost disappeared as the concentration of PEDOT: PSS was increased to 0.05 wt%. This can be explained by the fact that, the PEDOT:PSS filled the nanoslots unlike 20 nm gap structures where it remains only on the top of the nanogap and has been shown experimentally by Geunchang *et al*. for MXenes^29^. Microscopically, the field enhancement is explained in terms of accumulation of surface charges near the aperture edges in the presence of incident THz^34^. When the slot is filled with the conducting PEDOT: PSS, it is expected that it establishes the connection between the two edges of the slot antenna and prohibits accumulation of the charge and hence destroying the resonant transmission. This can also be confirmed from the measurements for slots coated with EG doped PEDOT: PSS, where the THz transmission was almost diminished (figure 3b).

Figure 3(c) shows the calculated transmission depth for 200 nm slot antenna. The transmission depth was found to be ~85% and ~90% for 1.3 wt% PEDOT: PSS and EG doped PEDOT: PSS coated nanoslot antenna respectively. We also measured the transmission of PEDOT: PSS with 500 nm and 1000 nm slot antenna and the results are shown in figure 4. Figure 4(a,b) are the transmission spectrum and calculated transmission depth for 500 nm slot antenna respectively and 4(c) and 4(d) are corresponding results for 1000 nm slot antenna. It can be seen that these results are analogous to the 200 nm slot antenna.

**Figure 5:**
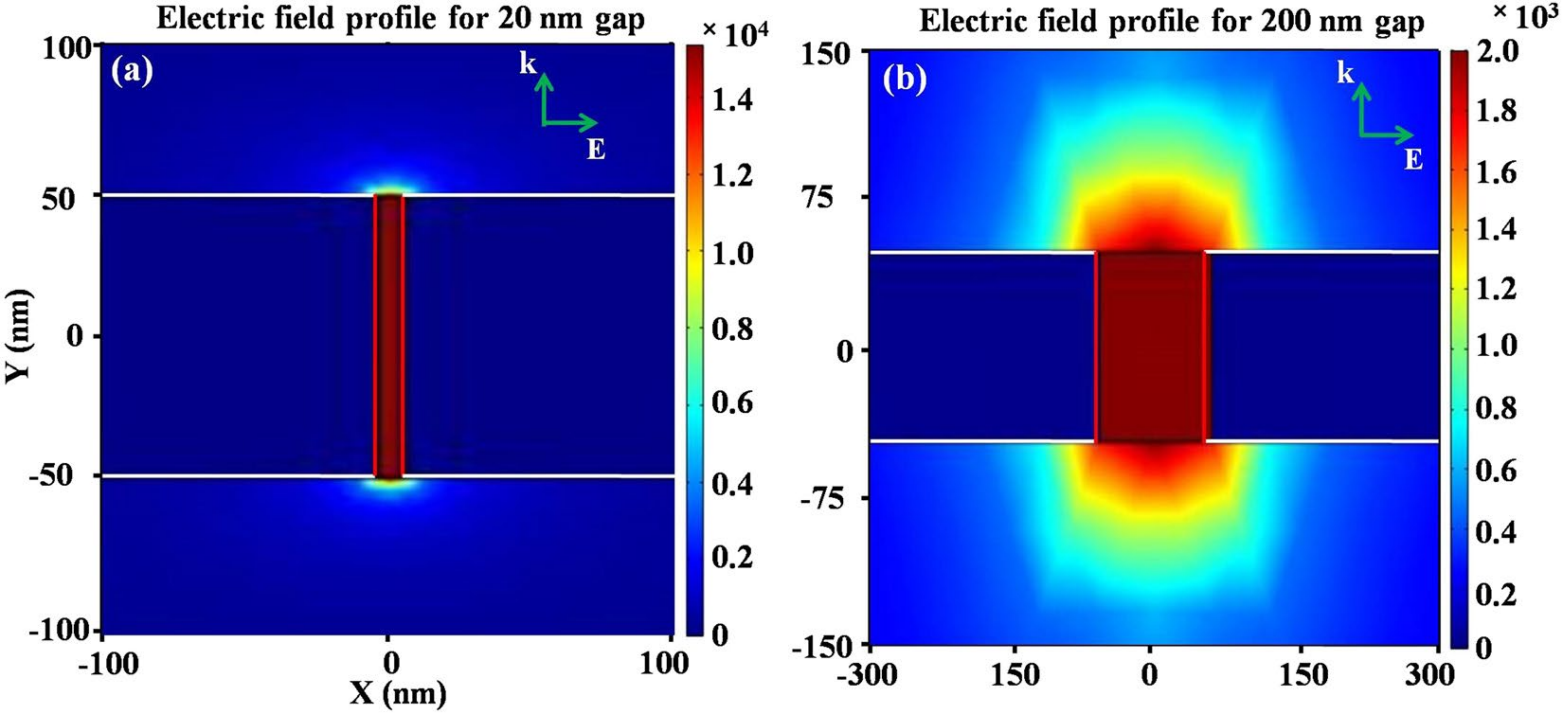
Electric field profile (V/m) of (**a**) 20 nm Al_2_O_3_ gap metamaterial structure and **(b**) 200 nm slot antenna. The field enhancement for 200 nm gap is nearly one order of magnitude lower than that of 20 nm gap, however the effective interaction depth of near field above the gap is higher in case of 200 nm gap.

To get further insight, we performed 2D finite element method (FEM) simulations implemented in COMSOL Multiphysics 5.3 to analyze the electric field profile for 20 nm Al_2_O_3_ gap and 200 nm air gap, as shown in figure 5(a,b). A periodic boundary condition was employed in X-direction and input and output ports were defined in the Y-direction. Physics controlled extra fine triangular mesh size was employed to obtain a convergent solution. The frequency dependent dielectric property of gold was defined from Drude model reported by Seo *et al*.^28^. Compared to 200 nm gap width, 20 nm gap shows nearly one order higher field enhancement, however the near field interaction depth on top of 20 nm gap is lower than that of 200 nm gap width. The near field interaction with PEDOT:PSS on top of the gap increases the sensitivity of the index mismatch and lead to the decreased transmission^35^ and resonance shift. In case of slot antenna, we expect that PEDOT:PSS was filled inside the gap^29^ and interacts with the enhanced electric field throughout the full propagation length. Also due to its conducting nature, it disrupts the accumulation of charges in the gap region and hence destroying the resonant transmission.

**Figure 6:**
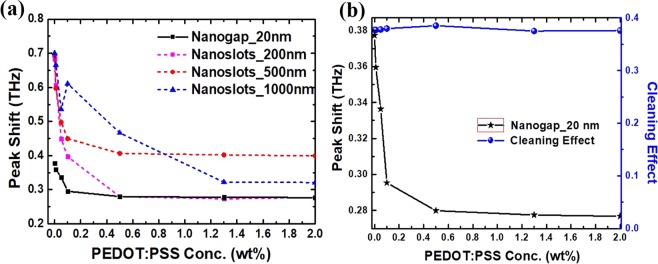
(**a**) Resonance peak shift of nano-metamaterials for different concentrations of PEDOT:PSS and (**b**) washing effect of the nano-metamaterial, which indicates that the original response is restored.

The shift in resonance frequency for different concentration of PEDOT:PSS coating was calculated as shown in figure 6(a). The resonance frequency shift was found to be smaller for 20 nm gap nano-metamaterial structure and can be attributed to the small interaction volume of PEDOT:PSS with the THz wave. However for large naogap metamaterial structures, this interaction volume increases to long length in PEDOT:PSS thickness (figure 5b), hence the resonance peak shift is in the broad frequency range. Also to check the repeatability of the device, we performed the THz transmission measurement for all the nano-metamaterial structures after washing off PEDOT:PSS and the results are shown in figure 6(b) for 20 nm nanogap structure. It was observed that the resonance frequency and THz amplitude of the nano-metamaterials are restored after washing the PEDOT:PSS film. The results indicate that this method can substantially restore the performance of the nano-metamaterial structures for other applications.

## Conclusion

In a nutshell, we have demonstrated effective control of THz transmission of PEDOT:PSS with different resonant nano-metamaterial structures along with tuning of resonance frequency. Pristine PEDOT:PSS was diluted with DI water to obtain various concentrations of it and its thin film on the nano-metamaterials were formed by drop-casting 10 μL of aqueous solution and annealing at 150 °C. The nano-metamaterials in combination with PEDOT: PSS thin films show significant reduction in transmission and change in resonance frequency with increasing the concentration. A transmission depth of greater that 60% and 85% were obtained for 20 nm nanogap structure and nanoslot antennas respectively along with red shift in resonance frequency. The pristine PEDOT: PSS (1.3 wt %) thickness was found to be ~455 nm and it is expected to be low for lower concentrations. The reduced transmission and change in resonance frequency can be related to the enhanced extinction coefficient of PEDOT:PSS due to strong localization of the THz field followed by field enhancement and increased conductivity. Further the performance of these nano-metamaterial structures can be restored by washing with hot DI water for other applications. Thus, a few 100 nm thin films of PEDOT:PSS can effectively control the resonant THz transmission and holds potential applications towards optical switching, modulation and other THz devices.
